# Autonomous wheelchair for patient’s transportation on healthcare institutions

**DOI:** 10.1007/s42452-021-04304-1

**Published:** 2021-02-22

**Authors:** André R. Baltazar, Marcelo R. Petry, Manuel F. Silva, António Paulo Moreira

**Affiliations:** 1 Departamento de Engenharia Eletrotécnica e de Computadores, Faculdade de Engenharia da, Universidade do Porto, Porto, Portugal; 2 CRIIS - Centro de Robótica Industrial e Sistemas Inteligentes, INESC TEC - Instituto de Engenharia de Sistemas e Computadores, Tecnologia e Ciência, Porto, Portugal; 3 Departamento de Engenharia Eletrotêcnica, Instituto Superior de Engenharia do Porto, Porto, Portugal

**Keywords:** Intelligent wheelchair, Mobility-on-demand, SONHO

## Abstract

The transport of patients from the inpatient service to the operating room is a recurrent task in a hospital routine. This task is repetitive, non-ergonomic, time consuming, and requires the labor of patient transporters. In this paper is presented a system, named Connected Driverless Wheelchair, that can receive transportation requests directly from the hospital information management system, pick up patients at their beds, navigate autonomously through different floors, avoid obstacles, communicate with elevators, and drop patients off at the designated operating room. As a result, a prototype capable of transporting patients autonomously in hospital environments was obtained. Although it was impossible to test the final developed system at the hospital as planned, due to the COVID-19 pandemic, the extensive tests conducted at the robotics laboratory facilities, and our previous experience in integrating mobile robots in hospitals, allowed to conclude that it is perfectly prepared for this integration to be carried out. The achieved results are relevant since this is a system that may be applied to support these types of tasks in the future, making the transport of patients more efficient (both from a cost and time perspective), without unpredictable delays and, in some cases, safer.

## Introduction

In hospital environments there are services where external consultations, examinations, analysis and surgeries are carried out involving the displacement of patients. This deserves some attention, especially for people with balance and mobility impairments associated with disabilities, pain, fractures or problems in lower limbs. Currently, the transportation of patients in hospitals is done by patient transporters, mostly non-specialized personnel. It could often be done using a wheelchair, and that would be more pleasant if done in an autonomous way. As a result, it would avoid delays and failures due to the lack of personnel to carry out the transportation.

In this context, and in the scope of a collaboration with the Portuguese Shared Services of the Health Ministry (Serviços Partilhados do Ministério da Saúde - SPMS), it was proposed the study and development of a system capable of transporting people. Given this idea, it was decided to develop this prototype according to the concept of an intelligent wheelchair, capable of safe autonomous navigation, offering an interaction with the patients, and communicating with the hospital information system, and other devices. It was chosen to implement this prototype in the transport of inpatients from the urology service to the operating room since, in this case, there is the possibility of the patient making the journey sitting on a chair instead of lying in bed.

Bearing these ideas in mind, in this paper it is presented the development of the Connected Driverless Wheelchair (CDW), an autonomous wheelchair capable of providing an on-demand mobility service to hospitals.

## Related works

Intelligent Wheelchairs (IW) are locomotion devices used to assist users with some kind of physical disability, where artificial control systems augment the user’s capabilities, reducing or eliminating the need for the user to drive [17]. They are usually controlled by a computer, apply several algorithms to derive meaningful information from sensors and act according to the user’s goals and the conditions of the environment. According to the literature, the main functions of an IW include [4, 9]:

- Multiple forms of interaction with the user (*i.e.* voice, hand, facial and head gestures);
- Autonomous navigation in changing environments;
- Communication with other devices (*i.e.* elevators, service robots, other wheelchairs).

Since the 1980s, a significant number of R&D projects addressed topics related to IW [7, 17].

One of the first IW projects was proposed by Madarasz *et al* in 1986 [11]. In 1994, Wellman et al [20] proposed a wheelchair equipped with two legs allowing for it to climb stairs and move on uneven terrain. Next year, Miller et al [12] developed Tin Man that allowed three operating alternatives: human guided with obstacle avoidance, move forward along a heading and move to a point (*x,y*). Later this system evolved to Tin Man II, presenting a decreased dependence on contact sensors, a modified user interface according to the needs of the community and an increase in the operating speed [12].

The prototype NavChair was developed at a later time, in the nineties [3, 18], based on an electric power wheelchair. The machine shared control with the user, was able to avoid obstacles and modified the user’s voice input command to achieve safe travel.

In 2007, Philips et al [14] proposed a control that worked according to the user’s need: the better the user dopes alone the less assistance he received. Basically, the user had full control of the wheelchair until he requested help. User input was collected through a brain-computer interface.

In 2008, Gao et al [8] placed a Light Detection And Ranging (LiDAR) sensor in a lift platform and evaluated the performance of the sensor for docking an IW system equipped with retro-reflectors.

As most wheelchair projects adopted complex hardware and software architectures, the IntellWheels project [5] focused in the development of an IW with a flexible and multimodal interface whose integration in wheelchairs available on the market could be done with few modifications and with a good target price [13].

The Autonomous Vehicle team of an alliance between a research group of the National University of Singapore (NUS) and the Massachusetts Institute of Technology (MIT) tested a self-driving wheelchair in Change General Hospital in 2016 [19]. It used the same technology that they developed for autonomous driving and all the user had to do was point where he wants to go and the wheelchair would drive him autonomously. The vehicle used three LiDAR to detect obstacles and to navigate. However, this sensor is expensive and using three in a wheelchair results in a very expensive prototype.

In 2017, a Wheelchair Mounted Robotic Arms (WMRA) was developed with the objective to make it autonomous, both at the level of localization and at object manipulation [16].

With the objetive to assist quadriplegic, paralyzed and handicaped patients who cannot drive a wheelchair by using a joystick, an IW was developed with a combination of two distinct ways of control: voice and head tilt [1].

More recently, in 2019, an IW was developed with the aim of providing mobility assistance for elderly people [6]. It was developed a prototype with some safety precautions and human interactive facilities. At the end, the authors conducted a user study that showed that the majority of the users preferred the developed IW over the conventional wheelchair.

WHILL [21] is a recent example of a company in this area with their Personal Electric Vehicles. They started by offering two different models of electric wheelchairs, one more portable that can be transported in any vehicle and another more robust that can be used in indoor and outdoor environments. Nowadays, they have an Autonomous Service that can be accommodated to the needs of the customers.

Although several prototypes with different approaches for an IW have been developed, none has been proposed that can communicate with a hospital information system and provide an on-demand mobility service.

## Proposed architecture

The proposed architecture for this prototype [2] presents an approach to integrate a driverless wheelchair (named Connected Driverless Wheelchair (CDW) in the scope of this project) in a hospital environment.

The original wheelchair structure has two motors (to which two encoders, essential for correct speed control and odometry, have been coupled directly in the motor shafts) and a joystick. A motor driver was chosen to control the speed of the wheels. Environmental sensing is achieved through a laser scanner and a Human-Machine Interface (HMI) displays real-time information and allows local interaction.

The software architecture is based on the Robotic Operating System (ROS) and all ROS nodes were developed in C++. This architecture can be divided in four parts: (i) hardware, involving all ROS packages needed to interact with the hardware; (ii) navigation, so that the CDW can represent the environment (as will be explained in Sect. 5), determine its own pose and execute a trajectory towards its goal; (iii) decision, consisting on the wheelchair’s ability to determine a trajectory it has to perform to transport a given patient to the operating room; and (iv) system interface, allowing information flow between the wheelchair and external systems.

The most important aspect of this architecture is the integration with the Hospital Information System (SONHO), performed through the exchange of HL7 messages, which defines how information is packaged and communicated from one party to another. SONHO is the base system of the Portuguese public hospitals, providing information about the patients (such as patient number, name, age, contact details, clinical history, among others) to other health information systems. With this exchange of messages it is possible to carry out the process of transporting a patient, from the time the request is made to the CDW until it arrives with the patient at the destination.

Finally, it is necessary to integrate the CDW with the elevators because the inpatient service and the operating rooms are often located on different floors of the hospital building. In partnership with Hospital de Santo António - Centro Hopitalar do Porto (CHPORTO), one of the two main public hospitals in Porto, and the company providing maintenance to their elevators, it was defined the protocol that encompasses the communication sequence between CDW and the elevator. In this integration is used a method already tested in a previous work [15], in which was installed an Ethernet I/O Module connected to the elevator PLC.

## Implementation

### Hardware

**Fig. 1:**
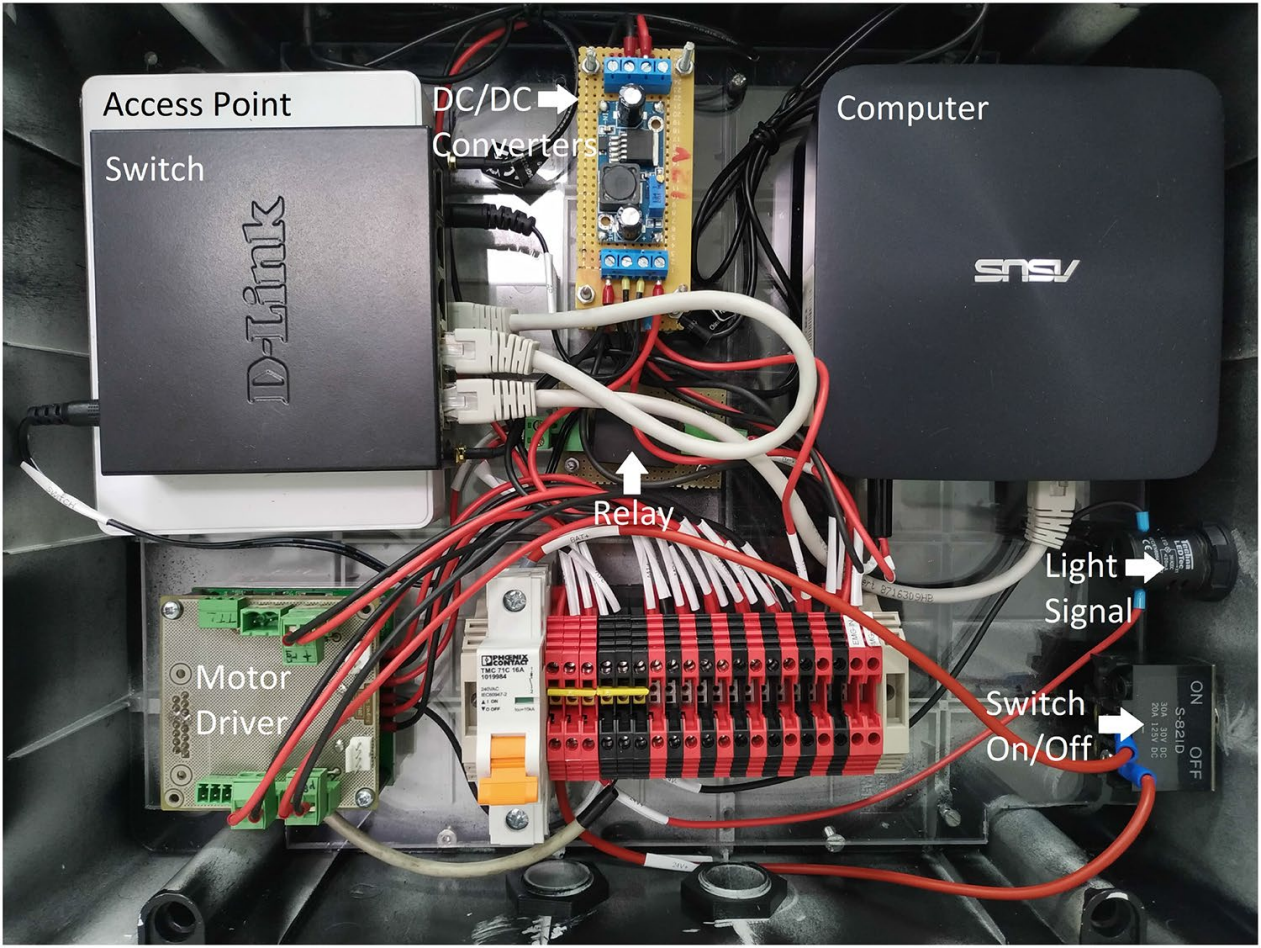
Electric enclosure with electronics inside

An electric enclosure of dimensions 390 $\times$ 310 $\times$ 130 mm was chosen to place most of the hardware (Fig. 1). Within the box it is the motor driver (Roboteq SDC2130) with a 32-bit microprocessor that runs the algorithms used to control wheel speeds in a closed loop, the Ethernet switch, the access point, the computer fitted with an Intel Core i5-4210U Central Processing Unit (CPU) running at 1.70 GHz, with 3.0 GB Random Access Memory (RAM), and a 256 GB Solid-State Drive (SSD) and three direct current to direct current (DC/DC) converters stacked to take up less space. Additionally, there are also several terminal blocks to facilitate connections to the outside, a circuit breaker to protect all components, a switch on the outside to turn on/off the entire system, a light signal that shows that the system is on and a relay activated by an emergency button. With this relay it is possible to cut off the power supply to the motors without interfering with the rest of the system.

The system is powered at 24 V, coming from a series of two 12 V batteries that are at the bottom of the chair. However, not all components support 24 V, hence the need to use DC/DC converters. These provide 5 V for the Raspberry Pi and the Ethernet Switch, 12 V for the LiDAR sensor and the access point, and 19 V for the computer.

**Fig. 2:**
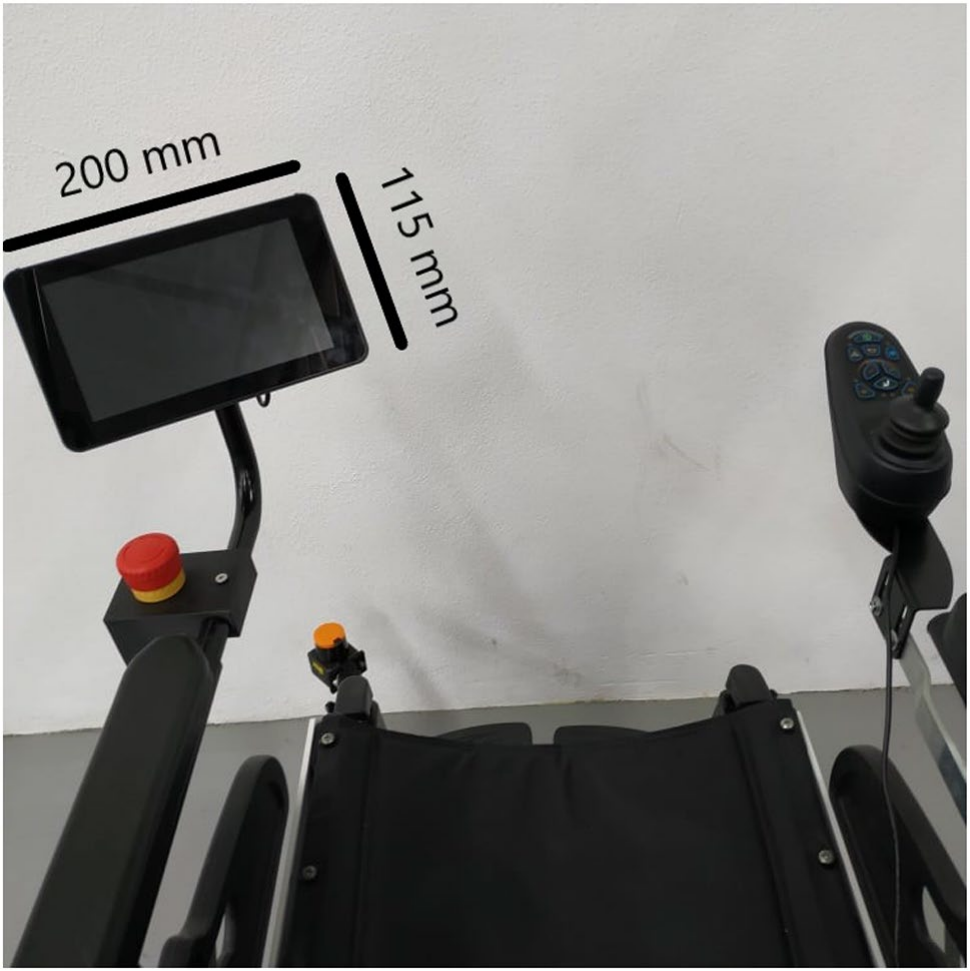
Patient’s view while being transported

Figure 2 depicts the joystick (on the right) and the emergency button and the touch display (on the left). An Arduino Nano was placed inside the joystick. It reads the variation of the stick movement in two axes (*x* and *y*), and these values are translated into speed commands for the motors on the microcontroller.

The display (115 $\times$ 200 mm) implements the HMI that allows the interaction with the patient and with the person who helps with the transport. It is fixed with a 3D printed support, allowing its rotation so that both the patient and those who help with transport can easily view the display. Next to this interface is the emergency button, also fixed using a 3D printed support.

**Fig. 3:**
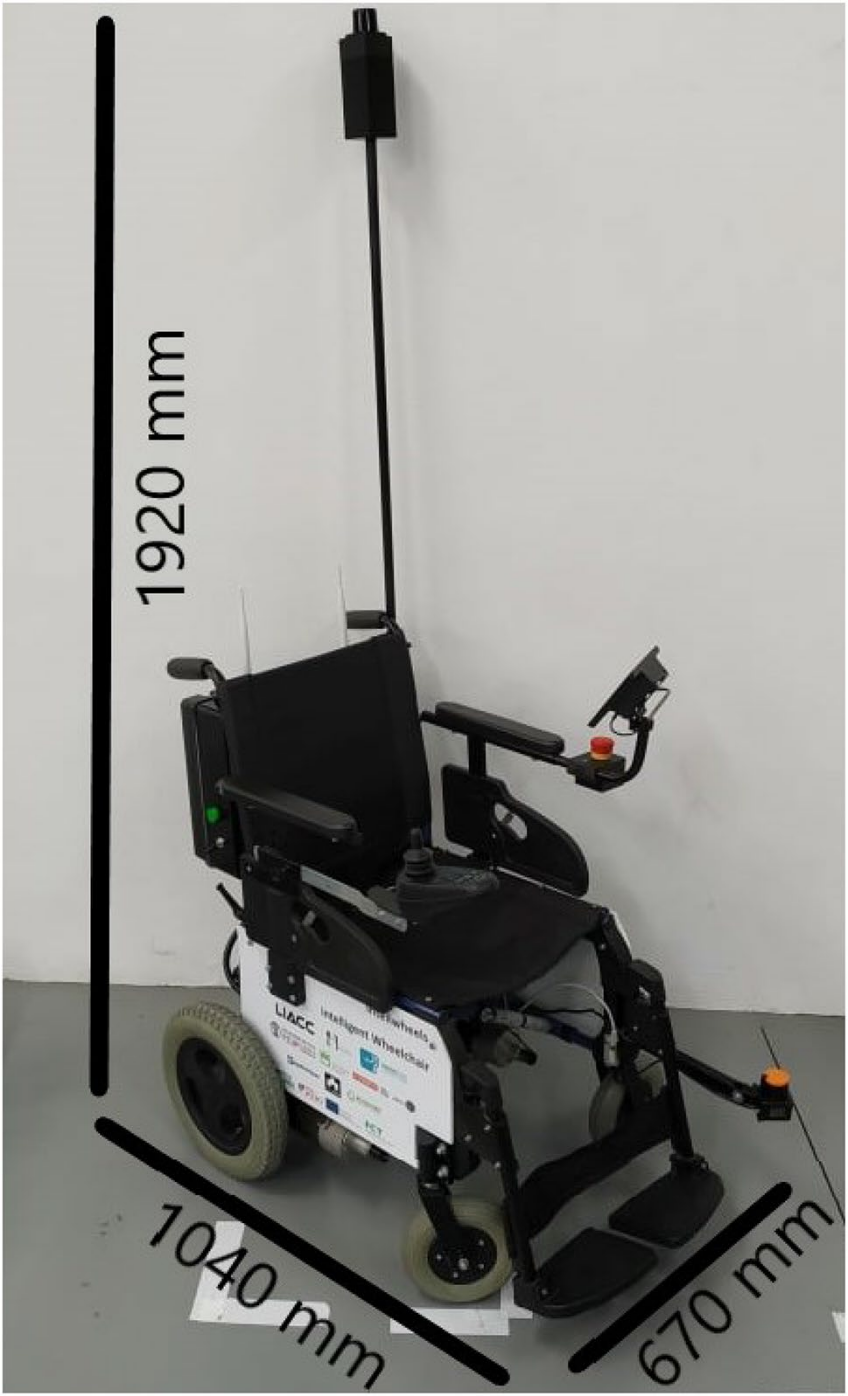
Final appearance of the wheelchair with an indication of its dimensions

It was decided to use two LiDAR sensors for environment sensing: one, with a maximum range of 20 m, was placed at a higher place, where there are not many dynamic obstacles, being used for navigation; another, with a max range of 10 m, was placed at a lower level to detect obstacles. The final aspect was taken into account, namely in the use of the black color, whenever possible, in cable routing through non-visible spaces and in the placement of components inside an electric enclosure in order to avoid an unpleasant appearance (Fig. 3). This figure also presents the wheelchair dimensions after the modifications. The system thus obtained resembles an electric wheelchair.

### Software

Taking into account the situation experienced during the development of this work, due to the COVID-19 pandemic, it was not possible to perform the actual integration in the hospital facilities. In this way, it was created a HL7 server that simulates the hospital system and were also created some messages that emulate the exchange of information between the CDW and the hospital information system (in the real scenario, these messages should have been added to the hospital information system, by the SPMS team, in the scope of this project).

Next are presented the steps followed to comply with the software architecture previously proposed. Initially is provided an explanation of the methodology used to read the values of the joystick and integrate them into the rest of the system. After is shown the entire configuration that needs to be carried out in the Instituto de Engenharia de Sistemas e Computadores, Tecnologia e Ciência (INESC TEC) Navigation Stack so that the robot can take advantage of this tool. Finally comes what would be used to integrate this system with the hospital system, namely, a HL7 client and the HMI on the robot, a HL7 server that would be running on a hospital computer and the hospital interface which allows the user in the operating room to generate the request to pick up a patient.

#### Navigation stack

INESC TEC’s Navigation Stack is the result of several years of development and testing. It has been developed on top of the ROS framework, and supports multiple traction modes, including differential, contains localization algorithms, drivers to interface with the robot hardware, a path planner, a controller for parametric trajectories and a map server.

The use of the Navigation Stack involves some requirements in the configuration of the robot, namely the definition of coordinate frames, the configuration of odometry source, the communication with motors drivers and the publication of the information received from sensors. The robot configuration is done by assigning values to certain parameters that are available in the different configuration files.

The definition of coordinate frames is done using the ROS tf package, which specifies their relationships and keeps them updated throughout the operation. Five coordinate frames are used: *map*, that represents the global coordinate frame, in which the robot will locate itself; *odom*, whose origin coincides with the robot initial pose and from which the robot movement is calculated through odometry; *base_link*, that consists of the reference of the robot, its relation with the odom corresponding to its movement; *laserNav*, corresponding to the LiDAR sensor mounted on the robot’s top that is used for the localization; *laser*, which corresponds to the LiDAR mounted in front of the robot. The odometry is obtained through the encoders attached to the motors. The Navigation Stack can communicate with the motors drivers, exchanging messages with the (linear and angular) speed commands and the encoder ticks.

Finally, it is necessary to configure the two LiDAR sensors, defining a static Internet Protocol (IP) address for each of them. The next phase consists of creating a 2-D occupancy grid map from laser and pose data collected by the CDW. In order for the robot to be able to locate itself, it uses an algorithm that compares the map initially made with the data provided by the LiDAR. The trajectories are built in a visualization tool, being composed by vertices and edges. The robot moves between different vertices through the edges.

#### Hospital information system

This subsection describes the exchange of messages between the CDW and the hospital information system - SONHO. The idea was to use the exchange of HL7 messages with SONHO to obtain the necessary data to carry out patient transportation. Some of the messages were already available but others were going to be developed by the SPMS team. However, due to the COVID-19 pandemic, the SPMS team was unable to develop the messages that would be necessary for this integration. Therefore, some messages had to be created, always taking into account the structure of the HL7 messages.

**Fig. 4:**
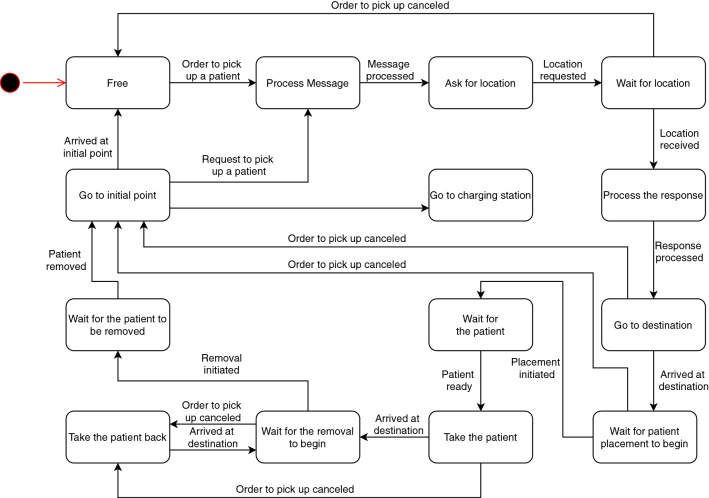
Connected driverless wheelchair state diagram: the red point indicates the initial state

A state diagram of the wheelchair has been defined, that deals with the exchange of messages and also with the movement of the robot (Fig. 4).

1. *Free*: Corresponds to the initial state, at which time the CDW is waiting to receive an order to pick up a patient.
2. *Process Message*: The CDW processes the request to pick up a patient through his Inpatient ID sent by the server and sends an acknowledgement to inform the server that the request has been accepted.
3. *Ask for location*: In this state is generated and sent a message where the inpatient ID obtained in the previous state will be used.
4. *Wait for location*: In this state the CDW is waiting for a reply to the message sent in the previous one. This reply contains the patient’s location so that it can be moved to a state in which this location is processed.
5. *Process response*: The purpose of this state is to get the necessary information from the message that contains the patient’s location.
6. *Go to destination*: With the patient’s location known, the next step is to go to pick it. This location has an associated vertex in the map generated by the navigation stack and, for the robot to go to it, it needs to call the service *GetPathToDestinationVertex*, where the value to pass is *destination_vertex*, that is the vertex where the CDW should go. After that, the trajectory must be monitored to know when the wheelchair reaches its destination. For that, it is necessary to subscribe the topic *path_percent_done*, where the percentage of the trajectory travelled is published.
7. *Wait for patient placement to begin*: After reaching the destination, the wheelchair waits for someone to inform that is has started to place the patient in it. This information will be given through the HMI.
8. *Wait for the patient*: At this state, the CDW is supposed to wait for the patient to be placed in it. There must be no movement on the part of the wheelchair as it may endanger the patient.
9. *Take the patient*: After receiving the signal, via the HMI, that the patient has been placed in the CDW, the next step is to take him to the destination using the same service and topic as in the “Going to destination” state.
10. *Wait for the removal to begin*: After reaching the destination with the patient, the wheelchair waits for someone to inform that it has started to remove the patient.
11. *Wait for the patient to be removed*: At this state the CDW should wait as in the state “Waiting for the patient” and there should be no movement.
12. *Take the patient back*: If, for some reason, the order is canceled when the wheelchair was already taking the patient to the destination, or waiting for the patient removal to begin, it should take the patient back to its bed.
13. *Go to initial point*: After the patient is removed, the CDW must move to its starting point where it will wait for new orders. This situation must also occur if the order is cancelled when the wheelchair was still going to the place where it would pick up the patient or waiting for the patient’s placement to begin.

**Fig. 5:**
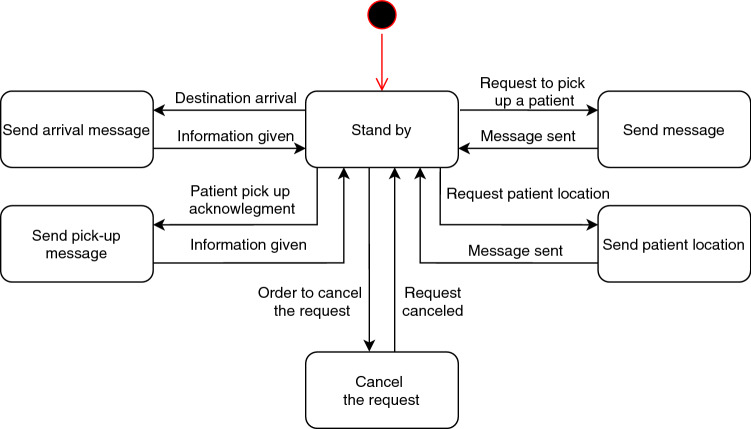
Server state diagram: the red point indicates the initial state

During this work, it was not possible to interact with the hospital information system, so a simulation of this system had to be developed. The solution consists of a HL7 server whose operation follows the state diagram shown in Fig. 5.

1. *Stand by*: State in which the server is ready to handle any message that can be sent by the wheelchair itself or through the interface managed by the hospital service (in this case, the message can be sent either by the interface itself or by a human).
2. *Request to pick up a patient*: When someone orders the wheelchair to pick up a patient it’s up to the server to request this directly to the CDW. The request is done by sending a message that will be processed by the CDW in the state “Processing Message”.
3. *Send patient location*: Sends the location of a specific patient to the wheelchair when requested. The location is sent in a message that will be processed by the client in the state “Processing response”.
4. *Send pick-up message*: When the server receives the robot’s acknowledgement for the pick up request, it communicates this information to the interface so that it can inform that the CDW has accepted the order.
5. *Send arrival message*: When the server is informed that the CDW has reached its destination it also communicates this to the interface.
6. *Cancel the request*: If the order has to be canceled at any time, the server will receive this information through the interface and send it to the wheelchair.

#### Human-machine interface

The HMI allows the exchange of information between the system, the patient, and the helping person. This HMI was developed to have an intuitive use and to provide the information that makes this process easier and more pleasant [10].

For this application to be able to communicate with ROS nodes, and thus interact with the software presented in the previous subsections, Rosbridge was used. On the HMI side, a TCP connection is established. On the ROS side, it is launched the Rosbridge server.

**Fig. 6:**
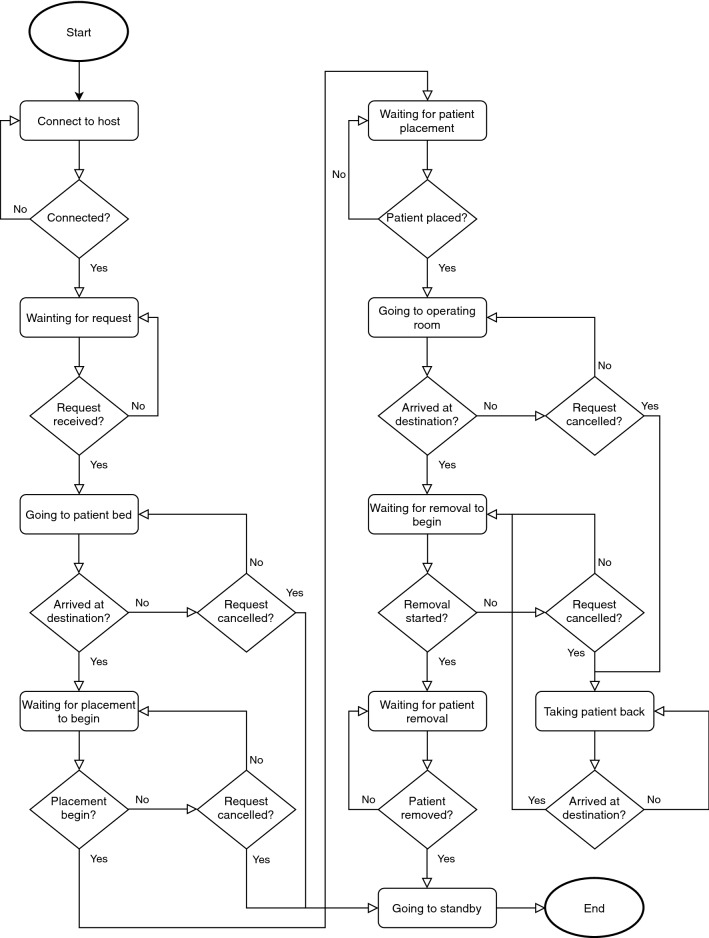
Human-machine interface flowchart

Through this server, the HMI obtains information about the CDW status and about the patient, namely, name, building, floor, room, bedroom and bed. The application also gives information about the action that was performed on it, namely when the buttons are pressed (Fig. 6).

**Fig. 7:**
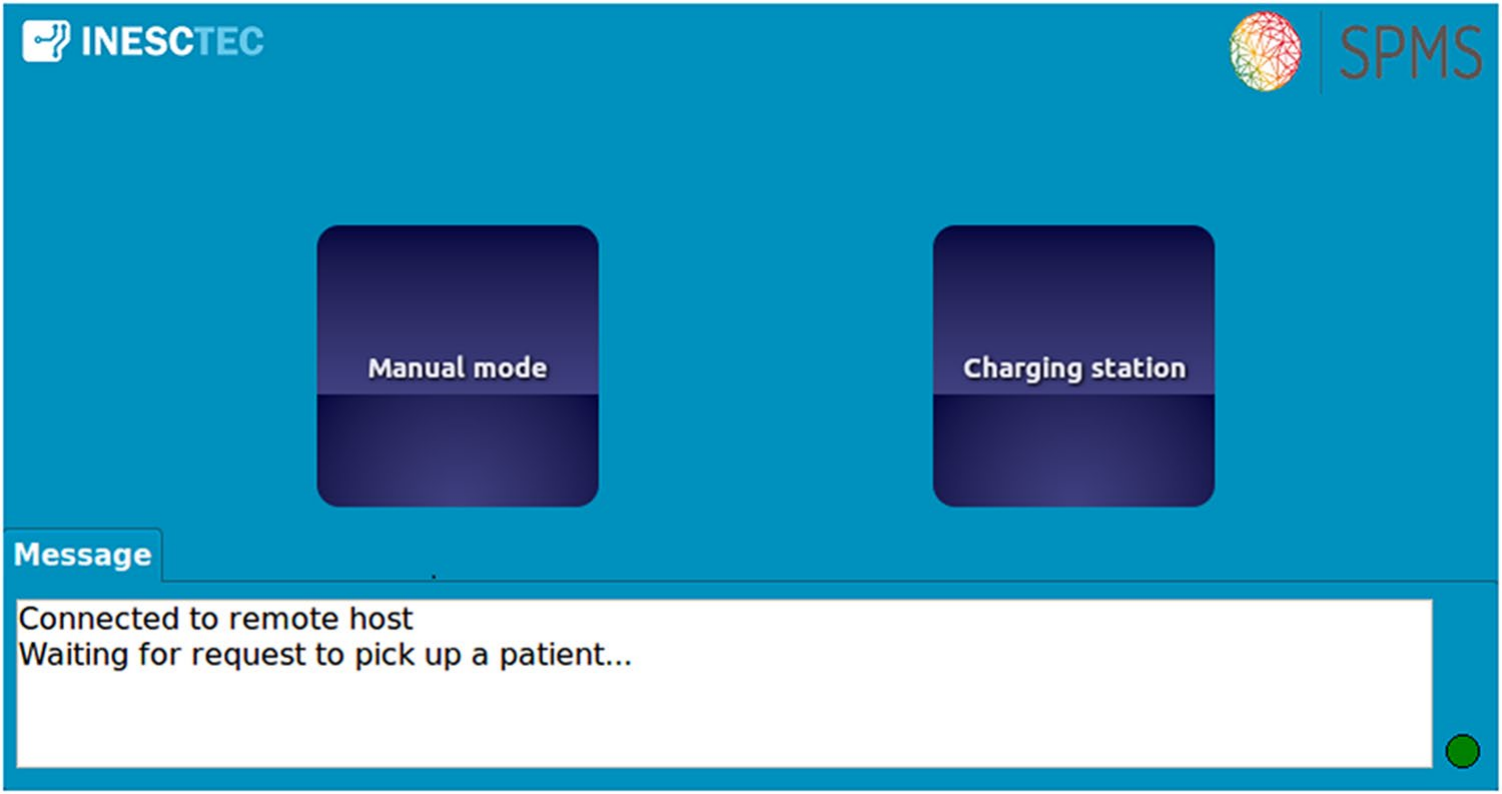
Initial state of the human-machine interface

The connection is performed automatically on application start. In the bottom right corner, there is a sign that shows whether the application is successfully connected (green color) or not (red color) (Fig. 7).

Once connected, the application is in a state in which it waits for a request to pick up a patient. At this time, it is still possible to switch to manual mode, using the “Manual Mode” button, or to send the robot to the charging station, using the “Charging Station” button. There is always a white box where relevant information about the status of the system is shown.

When the HMI receives a request to pick up a patient, the “Charging Station” button is hidden. The message box informs that the request has been received and presents some patient’s data, namely, the name and the location, where the wheelchair is going at this moment. The STOP button allows to stop the robot at any time: when it is pressed, it is replaced by the “Resume” button that allows to continue the order.

When the CDW arrives at its destination (the patient’s location), the “Start Patient Placement” button is shown in the place of the “Charging Station” one. The patient information is kept in the message box and the status of the CDW is shown: “Waiting for patient placement to begin”.

This way, the person helping to place the patient in the CDW must press the button before doing it, thus ensuring that the robot will not move during the process. After pressing the button, it is hidden and replaced by “Patient Placed”. This button should only be pressed when the patient is ready to be taken.

When the patient is ready, the CDW moves to the operating room. On the HMI, only the “Manual Mode” button is shown and the message box displays the patient’s name and the destination. When the wheelchair reaches the operating room the behavior of the HMI is similar to the one when the robot arrived at the patient’s bed. A “Start Patient Removal” button is shown, which must be pressed before starting to remove the patient. When this process is finished, the “Patient removed” button (which meanwhile appeared in place of “Start Patient Removal”) must be pressed to inform the CDW that it can return to the standby point. At this phase, the HMI returns to its initial state, waiting for a new request to pick up a patient.

The risks that may arise from a motion of the CDW while the patient is being transferred in or out of it are: (i) the fall of the patient when he is transferring himself alone in or out of the CDW; and also, (ii) the risk of the patient being hit by the CDW if it starts moving and the patient has no time to move out of its way. The adoption of the “Start Patient Placement” and “Start Patient Removal” buttons allows to guarantee that the wheelchair never moves during these phases, eliminating the above mentioned risks.

At any stage, the HMI offers the option of switching to “Manual Mode”: in this case the patient can manually control the CDW using the joystick. If this mode is activated, the color of the button changes from blue to orange to warn of the “dangers” that this mode involves.

#### Hospital interface

As mentioned, it was not possible to proceed with the integration in the hospital and several features had to be simulated. The hospital interface, which is the interface that exists in the operating room, through which the CDW would be requested, is part of these features. Thus, Lazarus [10] was used to develop this interface. It was also used a connection via TCP to the Rosbridge server.

The hospital interface receives information about the status of the CDW and, when necessary, the user of this interface makes a request to pick up a patient through his Inpatient ID.

**Fig. 8:**
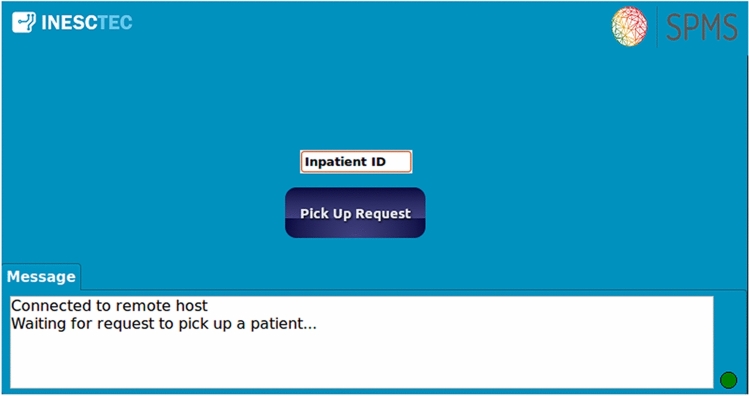
Hospital interface

As in the HMI, the connection is performed automatically on application start and there is the same signal in the bottom right corner that indicates the status of the connection (Fig. 8). At this moment, a text box is displayed where the Inpatient ID must be inserted, followed by pressing the “Pick up request” button to send the order. In this application, there is also a white box where relevant information about the status of the system is shown.

After sending the order, an acknowledgement is presented in the message box. Therefore, the request remains to be accepted by the CDW, which depends on whether the wheelchair is free to do so or not. When this feedback is provided, the robot starts processing the request, giving the option to cancel the order at any time.

In a normal situation (order not canceled) the interface will wait for the information indicating that the CDW has reached the operating room, and that it is waiting for the helping person to remove the patient. After receiving the information that the patient has been removed, the interface returns to its initial state and it is possible to make a new request.

## Results and discussion

**Fig. 9:**
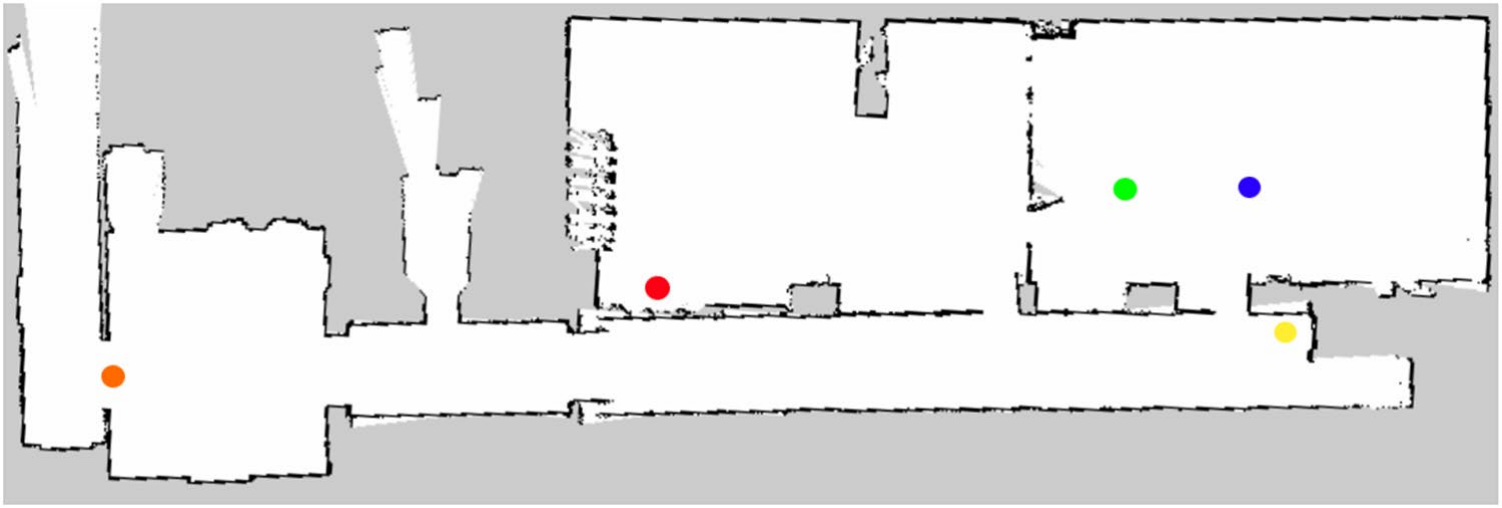
Map of the centre for robotics in industry and intelligent systems building and main points of interest: charging station (red), standby (yellow), operating room (orange), patient A (green) and patient B (blue)

Since it was not possible to apply the system in the hospital’s facilities, all the tests necessary for its validation were carried out in the Faculty of Engineering of the University of Porto (FEUP), more specifically in the facilities of the Centre for Robotics in Industry and Intelligent Systems (CRIIS). To carry out the tests in this environment, it was necessary to build the map shown in Fig. 9.

**Fig. 10:**
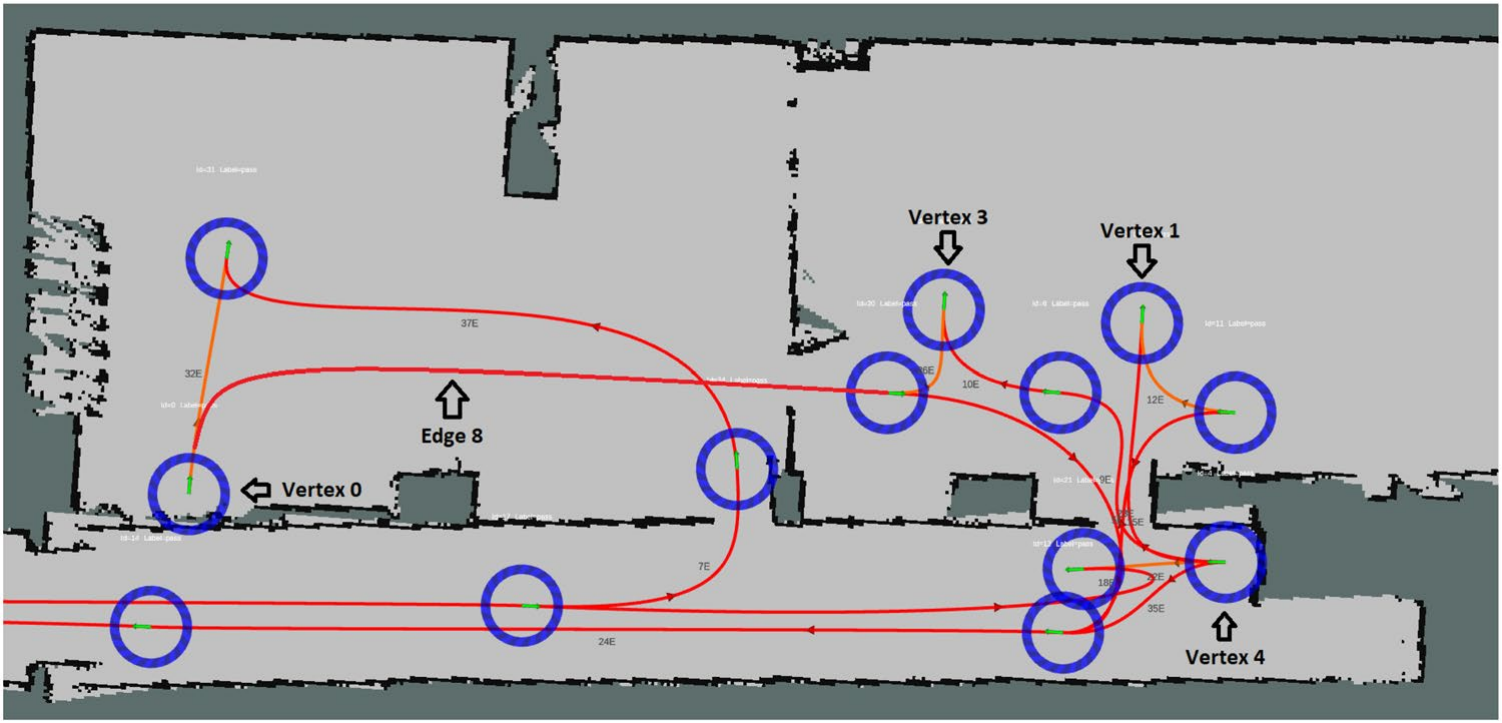
Trajectory of the CDW from charging station to patient location

In a first stage, the tests of the normal system operation were carried out. In this way, the prototype was placed in its charging station and an order was sent through the HMI. This request was correctly processed by the CDW and it went to the patient’s location. In Fig. 10 it is possible to see the starting and ending points, vertex 0 and 3, respectively, of this trajectory, in which the CDW traversed edge 8.

Upon reaching this location, the HMI assumed a state in which it waits for someone to press the “Start Placement” button. Once this button is pressed, the CDW informed that it was waiting for the placement to finish. After the patient was installed, the robot went to the operating room. When the CDW reached its destination, it was expected that someone would press the “Start Removal” button on the HMI and help the patient to get out of the wheelchair. Next the CDW went to the standby point (vertex 4). This way, the CDW completed a full task, waiting in the end for new orders.

In the sequel, some tests were performed to validate the CDW’s response to a second request. The first test consisted of performing the same task as before, but after the wheelchair was at the standby point, another request was sent to pick up a different patient. After receiving the order, the CDW went to vertex 1, thus making a shorter path compared to when it started at the charging station. Then, the behaviour was similar to that presented in the previous test, having successfully taken the patient to the operating room. The second test consisted of sending the second order when the CDW was returning to the standby point. The wheelchair reacted immediately to this request, without going to the standby point. Finally, to test this response to a second request, this was done when the CDW was taking another patient to the operating room, that is, it had not yet completed its task. When the order was received, nothing changed in the behaviour of the wheelchair, which concluded the transport to the operating room. The difference here was that once this transport was finished, the order already received was immediately processed and the CDW went straight to the patient location.

The wheelchair behaviour in case the order is cancelled is also very important. Two tests were performed to validate this: one in which the order was cancelled while the CDW was going to the patient and another in which the cancellation was made when the wheelchair was transporting the patient. In the first test, while the wheelchair was going to the patient’s bed, the order was cancelled and the CDW immediately returned to the standby point. In the second test, the order was cancelled when the wheelchair was transporting the patient to the operating room. The CDW went back to the place where the patient was picked up and waited for someone to remove the patient. Then, the CDW went to the standby point, waiting for new orders.

The most critical moments for cancelling the order are when the patient is being placed or removed from the wheelchair. As previously stated, if the system is not prepared for this, the robot may move and hurt the patient. To test this behaviour, the order was cancelled while the CDW was in these states and, in both cases, the CDW did not move. This is due to the existence of the “Start Placement” and “Start Removal” buttons, which place the system in a state where it completely ignores the cancellation orders, ensuring that the CDW does not perform any manoeuvre while the patient is being installed in or removed from the CDW.

An important component in the safety of the system is the emergency button, which can be pressed at any time by the patient or another person. Since the button only cuts off the power to the motors, it only makes sense to test the system’s behaviour when the robot is in motion. Therefore, a test was performed in which the button was clicked 10 times during a task: in all cases, the CDW stopped its movement immediately and only moved again when the button was released. This behaviour was also observed when using the STOP and RESUME buttons on the HMI.

To test the endurance of the system, a 9-hour test was carried out, during normal office hours (9:00 a.m.–18:00 p.m.). During the first eight hours was performed one sequence of requests per hour, each sequence being composed of 4 requests. In the last hour the system was left on standby, without performing any action. Each sequence was performed twice. During this test, and due to the situation caused by the COVID-19 pandemic, the facilities used for the tests had low occupancy of people, all doors were open, and only a few passers-by “presented” obstacles for the robot motion. Nonetheless, the CDW was able to identify all “obstacles” and react accordingly, by stopping its motion. This test demonstrated that the system can work for a whole day without presenting any anomaly.

Another important aspect of this system is its autonomy. To evaluate the performance of the CDW in this respect, the battery level has been measured every hour during the 9-hour test. The first measured value corresponds to the initial instant, before the CDW performs any task, and the following values were measured hourly, before the CDW started the next task sequence. The last measurement was made after the wheelchair remained an hour without performing any task. To make this test as realistic as possible, it was ensured that someone was always in the robot when it came to a part of the task in which the patient was being transported, as this additional weight influences the energy consumption of the motors. The battery level showed a total drop of 1.6 V, and an average drop of 0.17 V per hour. Bearing in mind that the minimum voltage value recommended by the manufacturer is 11.4 V per battery, at the end of the test there was still autonomy for about five and a half hours.

## Conclusions and future work

In this paper was presented the Connected Driverless Wheelchair (CDW), developed by a collective specification with SPMS and Hospital de Santo António - CHPORTO, and to be applied in the urology service of this hospital.

Based on the proposed architecture, all the necessary hardware was integrated. An electric enclosure was chosen to place most of this hardware to provide a good appearance of the CDW.

The software development was affected by the pandemic situation experienced during this work. It was thus necessary to simulate the HL7 client, so that the CDW could receive an answer to its requests to SONHO. At the same time, the INESC TEC Navigation Stack was also configured. This tool provided by CRIIS allowed the CDW to locate itself and to autonomously navigate in a previously mapped environment. After developing the software related to the HL7 messages and having the Navigation Stack configured, these two parts were integrated. Next was created an HMI providing information about the status of the system and the progress of the tasks. Another interface has been developed for the user in the operating room, allowing to send requests to the CDW. All these components were integrated into the final system that was tested in the premises of FEUP, in an environment that simulated the hospital.

In conclusion, it was verified that the CDW was able to locate and navigate autonomously on a known map, to communicate with the developed HL7 server, by correctly interpreting requests and requesting for the necessary information to carry out the transport, and to exchange information with the HMI and the hospital interface.

Concerning future work to be carried out with this CDW, the most important component will be the integration in the hospital, where there will be communication with SONHO. Furthermore, the scenario described in this paper only considers one wheelchair. In a real scenario, on which a fleet of CDW will be needed, the mobile robot coordination and scheduling software developed by INESC TEC, and presently used for managing fleets of autonomous robots used in industrial logistic applications, will be adapted to managing a fleet of CDW. Another aspect to improve is the strategy to avoid obstacles. Presently, when the CDW detects an obstacle it stops and waits for the path to be clear. However, a new functionality is currently being developed that will allow the CDW to deviate from obstacles that arise in its path. The integration with the elevators and automatic doors should also be tested, to allow the navigation between different floors.

Another interesting aspect to develop would be to improve the HMI, by adding features to entertain the patient, for example by presenting news or curiosities. This can distract the patient, reducing his anxiety during the transport. Finally, it would be interesting to conduct two surveys: one with the users of the system, to see how the transport can be made more pleasant, to understand the level of stress that some people may feel while being transported by an autonomous system and how to avoid it, and another with people who help with transport, in order to understand whether they prefer to do this type of work or other useful tasks in the hospital.
